# Orbital Implant Surgery with Costal Cartilage Graft Is Associated with Better Symmetry and Improved Cosmetic Appearance

**DOI:** 10.3390/jcm14062052

**Published:** 2025-03-18

**Authors:** Ushio Hanai, Yotaro Tsunoda, Hitoshi Nemoto, Yoshihiro Nakagawa, Takahiro Suzuki, Tadashi Akamatsu

**Affiliations:** 1 Department of Plastic and Reconstructive Surgery, Tokai University School of Medicine, Isehara 259-1143, Japan; y-tsunoda@tokai.ac.jp (Y.T.); nemoto.hitoshi.w@tokai.ac.jp (H.N.); at7071@tokai.ac.jp (T.A.); 2 Department of Ophthalmology, Tokai University School of Medicine, Isehara 259-1143, Japan; nakayoshi@tokai.ac.jp (Y.N.); taka-su@tokai.ac.jp (T.S.)

**Keywords:** orbital implant surgery, ocular prosthesis, costal cartilage graft, appearance, short-term outcomes, complications, additional surgery, orbital fracture, enophthalmos

## Abstract

**Background/Objectives**: In Japan, artificial orbital implants are not approved as medical materials, limiting the number of facilities that perform orbital implant surgery. However, this procedure is crucial for improving the quality of life of ocular prosthesis users by enhancing cosmetic outcomes. This study aimed to evaluate the short-term outcomes of orbital implant surgery using costal cartilage grafts and assess the cosmetic impact by comparing upper eyelid positions between patients who underwent the procedure and those who did not. **Methods**: Patients were divided into two groups: those who underwent evisceration and orbital implant grafting with costal cartilage (Group 1) and those who used a prosthetic eye without an orbital implant (Group 2). In Group 1 cases, following evisceration, a spherical implant was created using the sixth autologous costal cartilage and covered with four pedicled scleral flaps. The incidence of complications and the necessity for additional surgery were investigated through medical records, and both complications and upper eyelid symmetry were assessed at least 12 months after the final surgical procedure. **Results**: A total of 23 patients were included: 13 in Group 1 and 10 in Group 2. Group 1 had a significantly lower median age (52 vs. 68 years, *p* = 0.002) and a higher proportion of females (76.9% vs. 30%, *p* = 0.024). Upper eyelid asymmetry was significantly greater in Group 2 than in Group 1 (*p* < 0.05). Orbital fracture was associated with a higher risk of requiring additional surgery (100% vs. 37.5%, *p* = 0.075), though not statistically significant. **Conclusions**: Orbital implant surgery with costal cartilage grafts improves eyelid symmetry and cosmetic appearance. Early and accurate orbital volume repair is essential for preventing enophthalmos.

## 1. Introduction

When evisceration of the eyeball is performed due to trauma or ocular disease, the eyeball collapses. However, the volume of the ocular prosthesis alone is insufficient to compensate for the lost volume in the orbit, and enophthalmos develops on the affected side alongside the asymmetry of the upper eyelid, creating a cosmetically unnatural appearance. This is especially likely in patients with orbital fractures [1], as the intraorbital volume is enlarged by the fracture. Furthermore, in many cases, patients who have used a thick, heavy ocular prosthesis for a long period have difficulty wearing it because the socket changes over time owing to the load of the ocular prosthesis, causing it to fall out or cause pain. A wide range of materials can be used during orbital implant surgery [2,3].

However, in Japan, artificial materials such as acrylic or hydroxyapatite are no longer approved as medical materials. Consequently, autologous tissues, such as costal cartilage [4] must be harvested, processed, and grafted. Therefore, orbital implant surgery is only performed at a limited number of centers in Japan. This procedure can improve the quality of life of ocular prosthesis users and has significant advantages. Costal cartilage grafts are used as orbital implants instead of artificial materials after eyeball evisceration.

The purpose of this study was twofold: to report the short-term outcomes of the institution’s orbital implant surgery protocol and to clarify the cosmetic perspective of this procedure by comparing the positions of the upper eyelids of patients who underwent this procedure and those who did not. Based on these objectives, the following hypotheses are proposed:

1. The group that underwent cartilage grafting shows significantly less upper eyelid asymmetry;
2. In cases involving orbital fractures, additional surgery is often required due to enophthalmos or blepharoptosis resulting from increased orbital volume caused by insufficient orbital fracture reduction.

## 2. Materials and Methods

### 2.1. Study Design/Sample

The study population comprised patients with ocular prostheses reviewed at our Department of Plastic and Reconstructive Surgery between October 2019 and April 2023. Two groups were studied: Group 1, comprising patients who underwent evisceration of the eyeball and orbital implant graft with costal cartilage, and Group 2, comprising individuals who used a prosthetic eye without orbital implants. The ocular implant surgery in Group 1 was performed by a single plastic surgeon.

This study was approved by the Clinical Research Review Committee of Tokai University (approval number: 24R077-001 MH). Since this was a retrospective study conducted in an opt-out format, it was not necessary to obtain informed consent from all patients; however, informed consent was obtained from patients whose face photographs could be used.

### 2.2. Surgical Technique 

#### 2.2.1. Evisceration Procedure

This procedure is performed by an ophthalmologist. The palpebral conjunctiva is incised at the limbus, and Tenon’s capsule is dissected up to the insertion of the extraocular muscles to expose the sclera. The cornea is excised, and the ocular contents are removed. Hemostasis is achieved on the inner surface of the sclera [5]. In Group 2, following this step, the sclera and conjunctiva are each sutured closed using absorbable sutures. In Group 1, orbital implant surgery is subsequently performed by plastic surgeons.

#### 2.2.2. Orbital Implant Surgery

**Figure 1:**
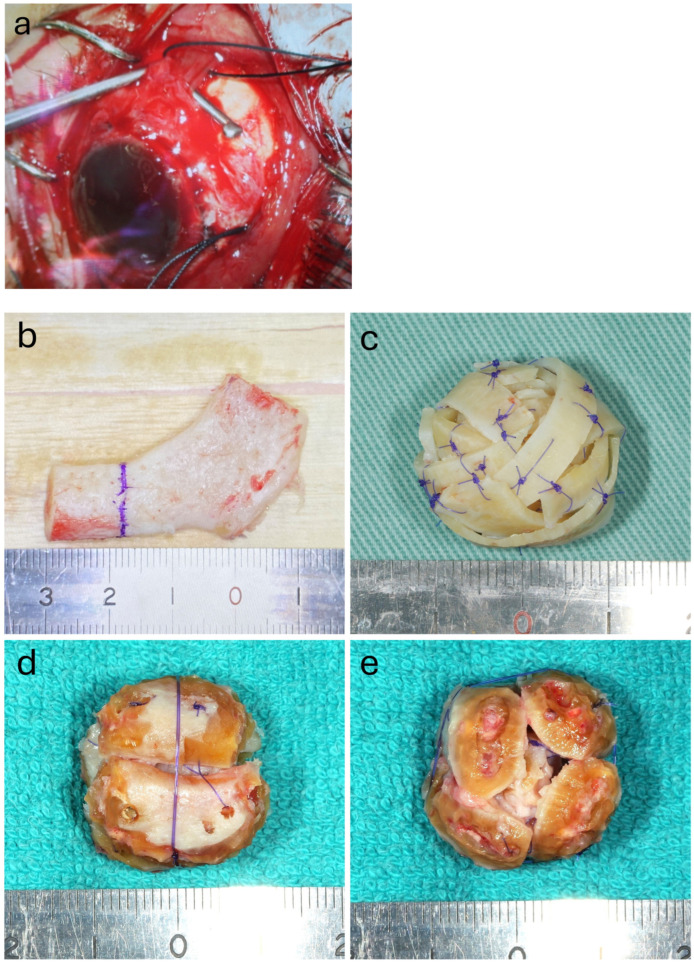

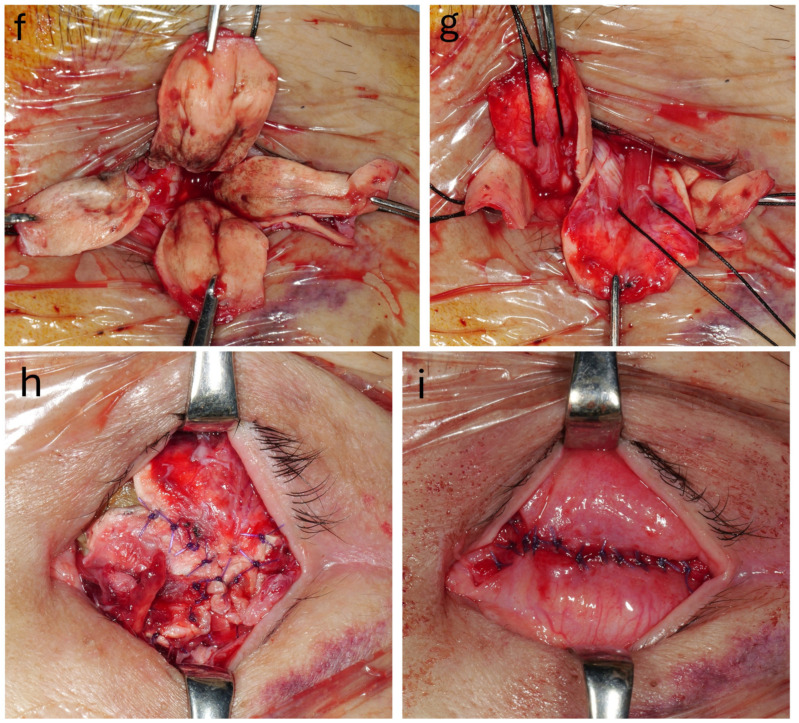
Intraoperative imaging showing various stages of the procedure and images of the spherical orbital implant. The superior rectus muscle is kept attached to the sclera using a silk thread as a landmark (**a**). The spherical orbital prosthetic implant can be made with a small volume of costal cartilage. The cartilage is divided at the marked area, using the left segment as the core (**b**). Strip-shaped pieces created from the right segment are then wrapped multiple times around the core to form a spherical implant (**c**). In elderly patients, the costal cartilage is highly calcified; hence, four blocks of cartilage are assembled, and then the surface is scraped with a motorized tool to fashion it into a sphere. In this case, the amount of rib cartilage to be harvested is greater (**d**,**e**). Four scleral flaps are shown unfolded. The posterior part of the sclera is detached around the optic nerve (**f**). Each rectus muscle serving as the pedicle of the flap (**g**). The scleral flaps are sutured together using absorbable thread (**h**) and the conjunctival sac is sutured (**i**).

The surgical technique followed the principles that have been published previously [6]. The first step involved the identification of the four external ocular muscles (superior, inferior, medial, and lateral rectus muscles) and evisceration of the eyeball by an ophthalmologist (Figure 1a). At the same time, plastic surgeons harvested costal cartilage through a skin incision on the right side of the chest. For females, the incision was made in line with the inframammary folds. The sixth costal cartilage was often used. The core cartilage piece and strips were then cut from the harvested costal cartilage (Figure 1b). The strips were wrapped around the core cartilage piece and sutured with absorbable threads to create a spherical cartilage ball (Figure 1c). The diameter of the prosthesis was approximately 20–23 mm. In elderly patients with severe costal cartilage calcification, it was not possible to cut or bend the strips; therefore, a bundle was made by combining large blocks, and the surrounding area was shaved to form a sphere (Figure 1d,e). This was followed by the creation of four scleral flaps using the extraocular muscles as pedicles (Figure 1), which were subsequently used to cover the costal cartilage ball implants inserted into the orbit (Figure 1f,g). The scleral flaps were sutured together using absorbable thread, and finally, the conjunctival sac was sutured (Figure 1h,i).

### 2.3. Postoperative Follow-Up

Postoperatively, patients were treated with antibiotic and steroid ophthalmic solutions for a period of two weeks. Following the resolution of postoperative tissue swelling, artificial eyes were fabricated at 4 to 6 weeks postoperatively for Group 1 and approximately 4 weeks postoperatively for Group 2. Custom-made artificial eyes made of polymethylmethacrylate (PMMA) were created for both Group 1 and Group 2. Postoperative follow-up assessments were conducted approximately every three months for a minimum of 12 months; subsequently, for cases without complications beyond this period, evaluations were performed approximately annually.

### 2.4. Variables

Data were collected on the following variables: age, sex, cause of blindness, preoperative complications/risks, and postoperative complications/additional surgery/symmetry of the upper eyelid protrusion. Upper eyelid protrusion was quantified using 3D images taken with VECTRA H2 imaging software version 7.4.6 (Canfield, Parsippany, NJ, USA) [7,8].

**Figure 2:**
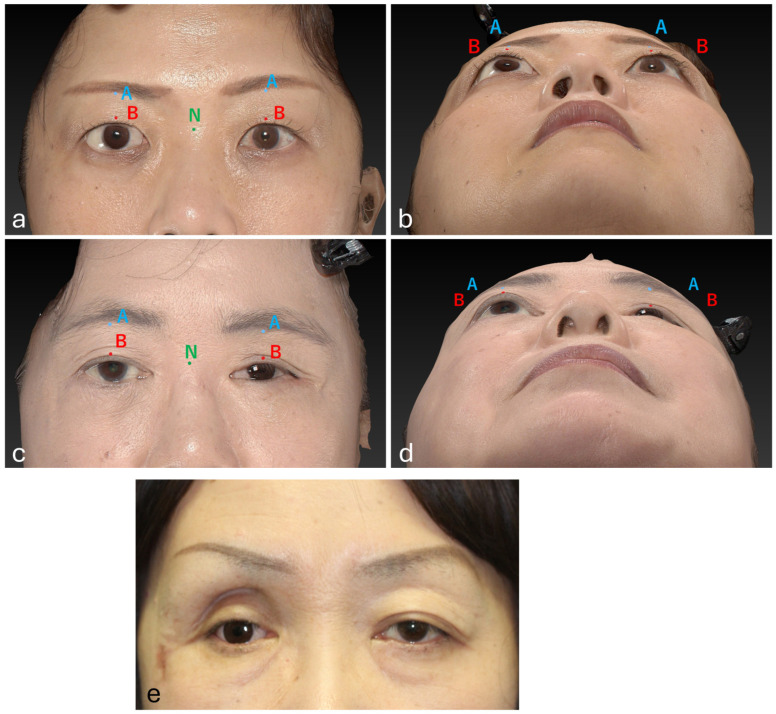
Imaging of patients from Groups 1 and 2. The measurement points were defined as the median point of the supraorbital rim (blue A), the median point of the upper eyelid margin (red B), and the most recessed point of the nasal root (green N). Exemplar imaging of a patient from Group 1 demonstrating the measurement points (**a**,**b**). Exemplar imaging of a patient from Group 2 demonstrating the measurement points (**c**,**d**). An exemplar case from Group 2 (**e**). The patient demonstrates a markedly sunken right supraorbital rim, which is conspicuous from the frontal view.

In this study, the anteroposterior positions of the two points on the upper eyelid (points A and B) were measured using the nasal radix (point N) as the reference point (Figure 2). Points A, B, and N were defined as the median point of the supraorbital rim, the median point of the upper eyelid margin, and the most recessed point of the nasal root, respectively.

### 2.5. Data Collection Methods

Data on demographic and clinical variables were collected by reviewing the medical records. The imaging-based measurements were performed using the VECTRA H2 imaging software version 7.4.6.

### 2.6. Standardization of 3D Imaging

All patients’ 3D images were captured by a single photographer with the patients in a seated position, in the same room, under identical lighting conditions, at the same distance, resolution, and settings.

### 2.7. Data Analysis

Mann–Whitney U tests and chi-square tests were used to compare Groups 1 and 2 on the above measures. Statistical analyses were performed by a professional biostatistician who was independent of the study team. All statistical analyses were performed using R version 4.1.3 (R Foundation for Statistical Computing, Vienna, Austria) following standard statistical guidelines, with a significance level of 0.05.

## 3. Results

### 3.1. Characteristics of the Study Population

The study population comprised 24 patients with ocular prostheses: 14 in Group 1 and 10 in Group 2. Out of the 14 cases in Group 1, one patient lacked postoperative 3D imaging data. Therefore, in Group 1, that one patient was excluded, and data from the remaining 13 patients were used for analysis.

The age distribution of the two study groups was significantly different. Group 1 had a significantly lower median age of 52 years (IQR: 35.50–62.50), whereas Group 2 had a median age of 68 years (IQR: 62.25–76.00), with a *p* value of 0.002, indicating statistical significance (Mann–Whitney U test). The sex distribution among the participants was significantly different between the two groups. In Group 1, three (23.1%) participants were male and 10 (76.9%) were female; however, in Group 2, three (30.0%) were female and seven (70.0%) were male, indicating a significant sex difference (chi-square test *p* value = 0.024).

Regarding the side of ocular prosthesis, Group 1 had an almost equal distribution between left and right sides, with six (46.2%) having a left ocular prosthesis and seven (53.8%) having a right ocular prosthesis. In Group 2, the left side was more prevalent (n = 8, 80.0%) than the right side (n = 2, 20.0%), although the difference between Groups 1 and 2 was not statistically significant (*p* = 0.099, chi-square test).

The causes of blindness varied among the participants. The leading cause in Group 1 was injury (n = 7, 53.8%), followed by congenital blindness (n = 2, 15.4%), diabetic retinopathy (n = 2, 15.4%), and keratitis (n = 2, 15.4%). In Group 2, injury was the predominant cause (n = 7, 70.0%), with additional cases due to cytomegalovirus (n = 1, 10.0%), septic endophthalmitis (n = 1, 10.0%), and thyroid eye disease (n = 1, 10.0%) (Table 1).

### 3.2. Upper Eyelid Measurement Differences Between Groups

A comparison of the upper eyelid measurements according to groups (1 and 2), measurement points (A and B), and sides (normal and affected) is presented in Table 2. The results showed no statistically significant differences in the Point A or Point B values between the normal and affected sides in both Groups 1 and 2, except for the Point A values on the affected side. These were significantly lower for Group 2 (median: −6.47, IQR: −15.08 to −4.96) than for Group 1 (median: −4.23, IQR: −6.28 to −2.59; *p* = 0.042, Mann–Whitney U test).

To further analyze the differences in Points A and B measurements between individuals in Groups 1 and 2, we compared upper eyelid asymmetry using the differences between the normal and affected sides for each case (Table 2).

At Point A, Group 1 had a median difference of 0.75 (IQR: −0.26–2.44), whereas Group 2 showed a more pronounced asymmetry with a median difference of 3.23 (IQR: 2.42–5.11). The associated *p* value of 0.003 (Mann–Whitney U test) indicated a statistically significant difference between the groups at Point A. Similarly, for Point B, Group 1 had a median difference of 0.86 (IQR: −0.53–1.78), and Group 2 had a median of 4.20 (IQR: 2.29–5.80), with a *p* value of 0.001 (Mann–Whitney U test), indicating a significant difference between the groups at this point. These results suggest that Group 2 exhibited more upper eyelid asymmetry than Group 1 at both Points A and B.

### 3.3. Complications and Additional Surgeries in Group 1

Preoperative complications in Group 1 included orbital fractures (n = 5, 38.5%), multiple facial fractures (n = 2, 15.4%), and bulbar atrophy (n = 1, 7.7%). The most common postoperative complication was isolated blepharoptosis (n = 3, 23.1%), with five (38.5%) reporting no complications (Table 3). Additionally, potential complications at the donor site of costal cartilage harvesting include pneumothorax due to pleural injury, hematoma formation, wound infection, and hypertrophic scars. However, no cases of these complications were observed.

Among Group 1 participants, additional surgeries were performed in eight (61.5%) cases, with blepharoptosis surgery and conjunctival sacroplasty being the most common procedures (n = 5, 38.5% each) (Table 4). All (100%) participants with orbital fractures required additional surgery, while only five (37.5%) of those without fractures required additional surgery, a difference that was not statistically significant (*p* = 0.075, Fisher’s exact test) (Table 5). The specific additional surgeries performed in the five patients with orbital fractures were as follows: blepharoptosis surgery (n = 4, 80%; levator aponeurosis tucking in three patients; frontalis suspension in one patient), conjunctival sacroplasty (n = 3, 60%), and revision surgery for chronic orbital fracture repair (n = 2, 40%).

### 3.4. Complications in Group 2

Postoperative complications in Group 2 included blepharoptosis (n = 5, 50.0%), enophthalmos (n = 6, 60.0%), narrow conjunctival sac (n = 6, 60.0%), lower eyelid malposition and laxity (n = 5, 50.0%), and ulceration (n = 2, 20.0%). In Group 2, there were no cases without complications (Table 6).

## 4. Discussion

### 4.1. Current Status of Orbital Implant Surgery

Orbital implant surgery is performed to compensate for the loss of orbital volume following enucleation, aiming to achieve both aesthetic and functional improvements. For patients who have unfortunately lost their vision, restoring a natural appearance is crucial for social reintegration. Internationally, artificial implants made of hydroxyapatite and porous polyethylene are widely used, as they have been reported to simplify surgical procedures while providing favorable aesthetic outcomes [9]. However, these artificial implants carry risks of complications such as exposure and infection. Previous studies have reported varying incidence rates of implant exposure and infection, ranging from 3% to 33%, depending on the materials used and the surgical techniques employed [9,10,11,12]. A study by Christmas et al. [9] analyzed 342 cases of artificial orbital implantation, reporting infection or implant exposure in seven cases (3%), while overall achieving satisfactory cosmetic results.

In Japan, however, artificial orbital implants have not been approved as medical materials, necessitating the use of autologous tissue in orbital implant surgery. Among the available options, autologous costal cartilage is a valuable material for maintaining long-term orbital volume, as it has a low risk of exposure and minimal susceptibility to absorption or deformation over time [13,14].

Nevertheless, costal cartilage grafting presents certain challenges. First, the procedure requires graft harvesting and shaping it into a spherical form, which extends the surgical duration by more than an hour. Second, donor-site complications such as scar formation, pneumothorax, and pain may occur. Third, in elderly patients with advanced calcification of costal cartilage, processing the graft can become difficult [6,15].

In this study, orbital implant surgery using costal cartilage was shown to improve the anteroposterior symmetry of the upper eyelid and enhance aesthetic satisfaction. Additionally, no cases of implant infection or exposure were observed, suggesting the safety of using autologous tissue. However, in cases involving severe trauma or preoperative severe ocular atrophy, volume augmentation alone was sometimes insufficient to achieve satisfactory outcomes, and additional procedures, such as conjunctival sacroplasty, were required. These findings highlight the need for a comprehensive treatment protocol for high-risk cases to optimize surgical outcomes.

### 4.2. Influence of Age and Gender on Surgical Selection

In this study, Group 1, which underwent orbital implant surgery, was significantly younger and had a higher proportion of female patients compared to Group 2, which did not undergo surgery. These findings suggest that age and gender may influence the decision to undergo orbital implant surgery. Generally, younger individuals and women are known to have a higher level of concern regarding their appearance and are more likely to seek aesthetic improvements. The results of this study likely reflect this trend. Conversely, older individuals, particularly men, may be more inclined to avoid surgery due to concerns about postoperative pain and complications. Additionally, since costal cartilage harvesting involves an additional surgical procedure, elderly patients may prefer to wear an ocular prosthesis alone to minimize surgical burden.

Although previous studies have provided limited evidence regarding the influence of gender on the selection of orbital implant surgery, the findings of this study suggest that both age and gender may play a role in surgical decision-making. Future research should involve a more diverse patient population to further analyze the impact of these factors in greater detail.

### 4.3. Measurement Methods and Evaluation

To objectively assess the effects of orbital implant surgery, the present study utilized the VECTRA 3D camera system to quantitatively measure the position of the upper eyelid. Traditionally, computed tomography (CT) imaging has been used to evaluate orbital fractures and enophthalmos; however, previous research has reported discrepancies between CT-based measurements of orbital fractures and clinical subjective evaluations [16]. This suggests that subjective assessments of facial symmetry may hold greater significance than CT-based measurements. Considering the emphasis on aesthetic satisfaction in this study, 3D imaging was employed instead of CT imaging.

The results demonstrated that orbital implant surgery significantly improved the symmetry of the upper eyelids. Notably, regarding Point A values on the affected side, which represents the deepest area of orbital depression, a significant difference was observed, indicating a more pronounced sunken-eye appearance in patients from Group 2. Sunken-eye deformity results from a deficiency in orbital volume [17], leading to enophthalmos and accentuating facial asymmetry (Figure 2e). This condition is a major aesthetic concern for affected patients, and the findings of this study suggest that orbital implant surgery may offer a viable solution for its correction.

### 4.4. Postoperative Complications

#### 4.4.1. Enophthalmos

Enophthalmos occurs when orbital volume replacement is insufficient following evisceration. In cases of severe trauma involving both globe rupture and orbital fractures, orbital contents are often damaged, leading to contraction over time and an increased risk of enophthalmos. Even with orbital implant surgery, achieving adequate volume replacement may be challenging in such cases [18]. In this study, two cases (15.4%) in Group 1 required additional orbital fracture repair. However, enophthalmos was observed in 60% of patients in Group 2, who did not undergo implant surgery, indicating that the procedure effectively prevented enophthalmos in the majority of cases.

#### 4.4.2. Blepharoptosis

Following evisceration, either true or pseudoptosis of the upper eyelid may develop. True blepharoptosis results from rupture of the levator aponeurosis, damage to the levator muscle itself, or impairment of its neural control [19]. In this study, one case of levator muscle rupture due to trauma was observed, which ultimately required a frontalis suspension procedure. In contrast, pseudoptosis is influenced by inadequate orbital implant volume or the shape of the prosthesis. If sufficient orbital volume is not maintained, the upper eyelid may descend, creating the appearance of ptosis. Adjustments to the prosthesis, such as increasing its vertical height or thickening its superior border, may help correct eyelid position. However, an excessively large prosthesis can reduce mobility and lead to lower eyelid malposition [20,21,22].

#### 4.4.3. Lower Eyelid Malposition and Laxity

An increase in the thickness and weight of an ocular prosthesis can contribute to lower eyelid laxity and positional abnormalities over time. In this study, 50% of patients in Group 2 exhibited lower eyelid malposition, raising concerns regarding the risk of future prosthesis displacement. To mitigate this issue, orbital implant surgery may be beneficial in reducing the burden on the lower eyelid by allowing for the use of a smaller and lighter prosthesis.

#### 4.4.4. Narrow Conjunctival Sac

A shortened conjunctival fornix due to orbital atrophy can lead to narrow conjunctival sac. In severe cases, prosthesis placement becomes difficult, necessitating mucosal grafting to reconstruct the socket. During evisceration, preserving as much of Tenon’s capsule and conjunctiva as possible is recommended [5]; however, in cases of severe globe rupture with orbital fractures, this is often unfeasible. In this study, conjunctival sacroplasty was required in four trauma cases and one case with severe preoperative ocular atrophy in Group 1 (38.5%). In patients with significant preoperative ocular atrophy, the scleral flap is generally sufficient to cover only the anterior surface of the costal cartilage implant, while conjunctival tissue remains insufficient, making secondary conjunctival sacroplasty necessary. Following mucosal grafting, complete engraftment and resolution of swelling require an additional one to two months. In cases where conjunctival sac contracture is anticipated, informing patients of the potential need for secondary surgery and establishing an appropriate treatment plan preoperatively is essential. In Group 2, conjunctival sac contracture was observed in 60% of patients (n = 6), who managed the condition through prosthetic adjustments. However, ulcer formation was noted in two cases (n = 2), raising concerns about progressive difficulty in prosthesis fitting, ultimately leading to aesthetic and functional issues.

### 4.5. Relationship Between Orbital Fractures and the Need for Additional Surgery

The causes of blindness among the study participants varied, but trauma was the most common etiology in both groups. This finding reflects the high number of trauma cases treated at the institution.

The results of this study did not show a statistically significant association between the presence of an orbital fracture and the necessity for additional surgery. However, all five cases in Group 1 with orbital fractures required additional procedures, suggesting that, from a clinical perspective, orbital fractures may be a significant risk factor for additional surgery.

Inadequate repair of orbital fractures increases the risk of complications such as enophthalmos and blepharoptosis. Therefore, in cases of orbital fractures accompanied by globe rupture, early and precise reduction of orbital volume is of critical importance [23,24]. In two cases where surgery was performed for chronic orbital fractures, severe scarring and contracture of the orbital contents, Tenon’s capsule, and conjunctiva were observed. Although enophthalmos was corrected, the upper fornix remained shallow, necessitating additional conjunctival sacroplasty. Furthermore, excessive overcorrection with an implant that is too large may impair ocular motility, emphasizing the need for a well-balanced surgical approach.

For cases involving orbital fractures, orbital implant surgery alone is often insufficient, and a comprehensive approach that includes fracture repair and conjunctival sacroplasty is essential to achieving optimal functional and aesthetic outcomes.

This study has some limitations. First, the sample size was small, and patients were recruited from a single center, which may limit the generalizability of our findings. Additional studies from multiple centers are required to confirm the findings of this study. Second, significant differences in age and sex existed between the two groups in this study. This is likely because the primary purpose of the surgery is cosmetic improvement, and younger patients, particularly females, are more inclined to undergo such procedures. In clinical practice, elderly patients, especially males, are less likely to request additional surgery, possibly due to concerns about post-operative pain and the small but real risk of pleural injury associated with costal cartilage harvesting. Additionally, previous studies in this field have not specifically addressed gender as a factor in aesthetic outcomes. Therefore, this natural selection bias should be considered when interpreting the results.

Third, potential bias in the allocation to Groups 1 and 2 exists, as the choice to undergo orbital implant surgery was not randomized but instead determined through agreement between the ophthalmologist and the patient. Therefore, biases may have influenced surgical decisions, with ophthalmologists likely guiding patients’ choices. Fourth, the decision to undergo additional surgery for complications is influenced by the patient’s wishes; therefore, it is not necessarily correlated with complication severity.

## 5. Conclusions

Orbital implant surgery using autologous costal cartilage has demonstrated significant improvements in upper eyelid symmetry and aesthetic satisfaction while maintaining a low risk of complications such as infection and implant exposure. The findings suggest that age and gender may influence surgical selection, with younger individuals and female patients being more likely to undergo the procedure. Although orbital fractures were not statistically associated with the need for additional surgery, all cases with fractures in the surgical group required secondary procedures, indicating their potential clinical relevance as a risk factor. Additionally, postoperative complications, including enophthalmos, blepharoptosis, lower eyelid malposition, and narrow conjunctival sac highlight the need for comprehensive treatment strategies, particularly in cases involving severe trauma or preoperative ocular atrophy. Given the limitations of this study, further multicenter research with larger sample sizes and randomized study designs is necessary to validate these findings and establish optimized treatment protocols for orbital volume reduction.

## Figures and Tables

**Table 1:** Causes of blindness among the study participants.

Causes of Blindness		Number	Percentage
Group 1	Congenital	2	15.4%
	Diabetic retinopathy	2	15.4%
	Injury	7	53.8%
	Keratitis	2	15.4%
Group 2	Cytomegalovirus	1	10%
	Injury	7	70%
	Septic endophthalmitis	1	10%
	Thyroid eye disease	1	10%

**Table 2:** Comparison of upper eyelid measurements between Groups 1 and 2.

Variables	Group 1 Median (IQR) (mm)	Group 2 Median (IQR) (mm)	*p* Value
Point A normal side	−3.84 (−5.84 to −2.92)	−4.02 (−7.50 to −2.67)	0.410 a
Point A affected side	−4.23 (−6.28 to −2.59)	−6.47 (−15.08 to −4.96)	0.042 * a
Point A difference in measurements	0.75 (−0.26 to 2.44)	3.23 (2.42 to 5.11)	0.003 * a
Point B normal side	−0.37 (−3.17 to 0.45)	0.10 (−2.31 to 2.08)	0.208 a
Point B affected side	−2.07 (−3.92 to −0.13)	−3.45 (−7.68 to −1.36)	0.284 a
Point B difference in measurements	0.86 (−0.53 to 1.78)	4.20 (2.29 to 5.80)	0.001 * a

* Significant *p* value; a Mann–Whitney U test.

**Table 3:** Pre- and post-operative complications in Group 1 participants.

Complications		Number	Percentage
Preoperative complications	Bulbar atrophy	1	7.7%
	Multiple facial fractures	2	15.4%
	Orbital fracture	4	30.8%
	Orbital fracture with levator palpebrae muscle injury	1	7.7%
	Absent	6	46.2%
Postoperative complications	Blepharoptosis	3	23.1%
	Enophthalmos and narrow conjunctival sac	1	7.7%
	Enophthalmos, narrow conjunctival sac, and traumatic blepharoptosis	1	7.7%
	Narrow conjunctival sac	2	15.4%
	Narrow conjunctival sac and blepharoptosis	1	7.7%
	Absent	5	38.5%

**Table 4:** Additional surgeries performed in Group 1 participants.

Additional Surgeries	Number	Percentage
Blepharoptosis surgery	3	23.1%
Conjunctival sacroplasty	2	15.4%
Conjunctival sacroplasty and blepharoptosis surgery	1	7.7%
Orbital fracture reduction and conjunctival sacroplasty	1	7.7%
Orbital fracture reduction, conjunctival sacroplasty, and blepharoptosis surgery	1	7.7%
Absent	5	38.5%

**Table 5:** Association between orbital fracture complications and rate of additional surgeries.

Orbital Fracture	Additional Surgery		*p* Value
	Not Performed	Performed	
	Number (%)	Number (%)	
Absent	5 (62.5)	3 (37.5)	0.075 b
Present	0 (0)	5 (100)	

b Fisher’s exact test.

**Table 6:** Postoperative complications in Group 2 participants.

Postoperative Complications	Number	Percentage
Blepharoptosis	5	50.0%
Enophthalmos	6	60.0%
Narrow conjunctival sac	6	60.0%
Lower Eyelid Malposition and Laxity	5	50.0%
Ulceration	2	20.0%
Absent	0	0%

## Data Availability

The raw data used for statistical analysis in this study are available from the corresponding author upon reasonable request.
